# Radiological patterns of pulmonary fungal infection in pediatric hematology and oncology patients

**DOI:** 10.1590/0100-3984.2021.0055

**Published:** 2022

**Authors:** Vera Bain, Anna Carlota Mott Galvão de Arruda Barrientos, Lisa Suzuki, Luiz Antonio Nunes de Oliveira, Nadia Litvinov, Karina Rodrigues Peron, Juliana Folloni Fernandes, Heloisa Helena de Sousa Marques

**Affiliations:** 1 Instituto da Criança do Hospital das Clínicas da Faculdade de Medicina da Universidade de São Paulo (ICr/HC-FMUSP), São Paulo, SP, Brazil.

**Keywords:** Mycoses/diagnostic imaging, Child, Adolescent, Immunocompromised host, Tomography, X-ray computed, Micoses/diagnóstico por imagem, Criança, Adolescente, Hospedeiro imunocomprometido, Tomografia computadorizada

## Abstract

**Objective:**

To describe the radiological findings in pediatric patients with hematological or oncological diseases who also have an invasive fungal infection (IFI).

**Materials and Methods:**

This was a retrospective study of all patients with IFI admitted to a pediatric hematology and oncology hospital in Brazil between 2008 and 2014. Clinical and demographic data were collected. Chest computed tomography (CT) scans of the patients were reviewed by two independent radiologists.

**Results:**

We evaluated the chest CT scans of 40 pediatric patients diagnosed with an IFI. Twenty-seven patients (67.5%) had nodules with the halo sign, seven (17.5%) had cavities, two (5.0%) had nodules without the halo sign, and seven (17.5%) had consolidation. The patients with the halo sign and cavities were older (123 vs. 77 months of age; *p* = 0.03) and had less severe disease (34% vs. 73%; *p* = 0.04). Ten patients had a proven IFI: with *Aspergillus* sp. (n = 4); with *Candida* sp. (n = 5); or with *Fusarium* sp. (n = 1).

**Conclusion:**

A diagnosis of IFI should be considered in children and adolescents with risk factors and abnormal CT scans, even if the imaging findings are nonspecific.

## Abstract (pt)

**Objetivo:**

O objetivo deste estudo é descrever os achados radiológicos de infecções fúngicas invasivas em crianças com doenças onco-hematológicas em um único centro, de acordo com a classificação antiga e a atual de imagens típicas e atípicas.

**Materiais e Métodos:**

Foram revisados os prontuários de todos os pacientes com infecção fúngica invasiva que foram internados em um hospital pediátrico de oncologia e hematologia de 2008 a 2014. Foram coletados dados clínicos e demográficos. As tomografias de tórax dos pacientes foram laudadas por dois radiologistas independentes.

**Resultados:**

Foram identificados 40 pacientes com infecção fúngica invasiva que realizaram tomografias de tórax. Vinte e sete pacientes apresentaram nódulos com sinal do halo (67,5%), sete tiveram cavitações (17,5%), dois tiveram nódulos sem halo (5,0%) e sete apresentaram consolidações (17,5%). Os pacientes que apresentavam achados de nódulos com sinal do halo e cavitações eram mais velhos (123 *versus* 77 meses; *p* = 0,03) e tinham menos sinais de doença grave (34% *versus* 73%; *p* = 0,04) do que os outros pacientes. Dez crianças apresentaram infecção confirmada (*Aspergillus* sp., n = 4; *Candida* sp., n = 5; *Fusarium* sp., n = 1).

**Conclusão:**

O diagnóstico de infecção fúngica invasiva deve ser considerado em crianças com fatores de risco e tomografias de tórax alteradas, mesmo que os achados das imagens sejam inespecíficos.

## INTRODUCTION

Invasive fungal infections (IFIs) are a major cause of morbidity and mortality in immunosuppressed adults and children^(1-4)^. Studies have shown that the observed incidence of IFI has increased in recent decades, probably due to improved survival among patients with hematological and oncological diseases, the greater use of immunosuppressive drugs, and improvements in diagnostic methods^(3,5)^.

The pathogens most often implicated in IFIs are *Aspergillus* sp. and *Candida* sp.^(1)^. The most common site of IFI is the lungs, which are affected in 60-80% of cases, followed by the sinuses, skin, liver, kidneys, eyes, and central nervous system^(5-9)^.

Making a definitive diagnosis of IFI in pediatric patients remains a challenge, because it usually requires invasive procedures such as bronchoscopy and biopsy, given that blood cultures have low diagnostic performance, especially for aspergillosis and other mold infections. It can be useful to determine the levels of the biomarkers galactomannan and beta-D-glucan, which have the same thresholds for adult and pediatric patients^(10)^.

Radiological findings are often nonspecific in children, and typical findings are more common in older children and adolescents^(8,11-13)^. There have been only a few case series describing the radiological findings of IFI in children^(6-9,11,12,14-17)^. The 2008 European Organisation for Research and Treatment of Cancer (EORTC) consensus classified the halo sign, cavities, and the air crescent sign as findings typical of IFI on computed tomography (CT) scans of the chest^(18)^. The revised 2020 consensus included nodules without the halo sign and consolidations as radiological patterns also indicative of IFI^(10)^.

The aim of this study was to describe the radiological findings of IFI in pediatric patients with hematological and oncological diseases, as defined in the previous and current EORTC guidelines^(10,18)^.

## MATERIALS AND METHODS

We reviewed the medical records of all patients with IFI admitted to a pediatric hematology and oncology hospital between 2008 and 2014. Patients for whom an antifungal agent had been prescribed were screened; those with risk factors for and signs/symptoms of IFI were included. Asymptomatic patients treated prophylatically with an antifungal agent were excluded. The study was approved by the Research Ethics Committee of the Hospital das Clínicas da Faculdade de Medicina da Universidade de São Paulo, in the city of São Paulo, Brazil.

We analyzed demographic and clinical characteristics (age, sex, and underlying disease); risk factors for IFI (neutropenia, corticosteroid and antibiotic use, presence of invasive devices, and mucositis); the clinical presentation; disease severity, as determined by admission to the intensive care unit and hemodynamic instability (hypotension for age or use of vasoactive drugs); diagnostic classification, as proven, probable, or possible according to the 2008 and 2020 EORTC criteria^(10,18)^; treatment (prophylaxis, drugs, and duration of therapy); and outcomes.

All CT scans were obtained with a commercially available 64-slice multidetector CT scanner (Brilliance; Philips Medical Systems, Best, the Netherlands). We employed model-based iterative reconstruction, and predefined protocols were selected according to patient age: tube voltage of 80-120 kVp; tube current of 80-120 mAs; slice thickness of 1.0 mm; and CT dose index of 0.94-7.24 mGy. No contrast was used. The CT scans were ordered at the discretion of the physician, in the following settings: during the investigation of an IFI; in patients with a prolonged fever of unknown cause; or during the follow-up of patients receiving antifungal treatment.

Images were interpreted by two radiologists, working independently, who were blinded to all clinical data. The interpretation was performed in three steps: in the first round, all CT scans were interpreted; in the second round, the CT scans for which there were differences in interpretation were reviewed by each of the radiologists separately, both of whom were blinded to the nature of the differences; in the third round, differences were resolved by consensus. The follow-up CT scans were also analyzed independently and classified as improved, unchanged, or worsened.

Descriptive analysis was performed for demographic data. Prevalence was expressed as absolute and relative frequencies. Continuous variables were compared by using the Mann-Whitney test, and categorical variables were compared by using the chi-square test or Fisher’s exact test, as appropriate. The level of agreement between the two radiologists was determined by calculating the kappa (κ) statistic. Statistical analyses were performed with the IBM SPSS Statistics software package, version 22.0 (IBM Corp., Armonk, NY, USA).

## RESULTS

We identified 73 patients ≤ 18 years of age with an IFI. Of those 73 patients, 42 (57.5%) had undergone chest CT. Two patients were excluded because the CT scans were technically unacceptable. Therefore, the final sample comprised 40 patients. We evaluated a total of 76 CT scans acquired from the selected patients. A CT scan had been obtained at the time of diagnosis in all 40 of the patients and during follow-up in 36. Levels of galactomannan and beta-D-glucan were not routinely determined at our hospital during the study period.

Of the 40 patients evaluated, 12 (30.0%) had acute lymphoblastic leukemia and 17 (42.5%) had acute myeloid leukemia. Of the 29 patients with leukemia, 13 (44.8%) were refractory to treatment or had relapsed. In our sample, the risk factors for IFI were as follows: use of broad-spectrum antibiotics, in all 40 patients; neutropenia, in 37 (92.5%); use of corticosteroids, in 11 (27.5%); chemotherapy, in 37 (92.5%); mucositis, in 29 (72.5%); and the presence of invasive devices, in 33 (82.5%). The following signs and symptoms were observed: fever, in 39 (97.5%); respiratory distress, in 22 (55.0%); pleuritic pain, in one (2.5%); skin lesions, in 1 (2.5%); and hemodynamic instability, in 12 (30.0%). Antifungal prophylaxis was used in 23 patients (57.5%). The median duration of treatment was 21 days. Of the 40 patients, 27 (67.5%) were treated with one antifungal agent and 13 (32.5%) were treated with two. Twenty-one patients (52.5%) recovered, five (12.5%) died of IFI, and 14 (35%) died of other causes or were lost to follow-up. Clinical and demographic data are shown in Table 1.

**Table 1:** Clinical and imaging characteristics of pediatric patients with CT findings considered typical or atypical of IFI in the 2008 EORTC guidelines^(18)^.

Characteristic	Chest CT findings		
	Typical (n = 29)	Atypical (n =11)	P-value
Sex, n (%)			0.48
Female	13 (45)	7 (64)	
Male	16 (55)	4 (36)	ND
Underlying condition, n (%)			
Acute lymphoblastic leukemia	9 (31)	3 (27)	
Acute myeloid leukemia	13 (45)	4 (36)	
Refractory to treatment or relapsed	11 (38)	2 (18)	
Autologous bone marrow transplant	2 (7)	1 (9)	
Allogeneic bone marrow transplant	1 (3)	1 (9)	
Other*	7 (24)	4 (36)	
Age (months), median	123	77	0.03
Age group, n (%)			ND
≤ 6 years	8 (2)	6 (55)	
7–12 years	9 (31)	5 (45)	
≥ 13 years	12 (41)	0	
Neutropenia, n (%)	28 (97)	9 (82)	0.17
Days with neutropenia before			
diagnosis, median	15	11	0.04
Broad-spectrum antibiotics, n (%)	29 (100)	11 (100)	ND
Corticosteroid therapy, n (%)	6 (21)	5 (45)	0.13
Chemotherapy, n (%)	28 (97)	9 (82)	0.07
Invasive devices, n (%)	23 (79)	10 (91)	0.65
Mucositis, n (%)	23 (79)	6 (55)	0.13
Signs and symptoms, n (%)			
Fever	29 (100)	10 (91)	0.27
Respiratory distress	16 (55)	6 (55)	1
Pleuritic pain	1 (3)	0 (0)	1
Hemodynamic instability	6 (21)	6 (55)	0.03
Skin lesions	0 (0)	1 (9)	0.27
Duration (days) of fever, median	9.0	5.5	0.30
Intensive care unit admission	10 (34)	8 (73)	0.04
Antifungal prophylaxis	17 (59)	6 (55)	ND
Fluconazole	2 (7)	1 (9)	
Itraconazole	7 (24)	0	
Voriconazole	3 (10)	1 (9)	
Micafungin	5 (17)	4 (36)	
Antifungal treatment			ND
Duration (days), median	21 days	22 days	0.60
Agent, n (%)			
Fluconazole	1 (3)	0	
Voriconazole	5 (17)	0	
Amphotericin B	12 (41)	9 (82)	
Amphotericin B + fluconazole	0	1 (9)	
Amphotericin B + voriconazole	11 (38)	1 (9)	
Outcome			0.59
Cure	14 (48)	7 (64)	
Death from IFI	3 (10)	2 (18)	
Death from other causes	9 (31)	2 (18)	
Loss to follow-up	3 (10)	0	
Chest CT findings^†^			
Halo sign	27 (93)	0 (0)	0.00
Consolidation	12 (41)	8 (72)	0.15
Cavity	7 (24)	0	0.15
Nodules	1 (3)	2 (18)	0.17
Ground-glass opacity	19 (65)	10 (91)	0.23
Pleural effusion	10 (34)	5 (45)	0.71
Other	24 (82)	8 (72)	0.66

* Neuroblastoma (n = 4); osteosarcoma (n = 2); astrocytoma (n = 1); hemangioendothelioma (n = 1); sarcoma (n = 1); pineoblastoma (n = 1); and retinoblastoma (n = 1).

† 1 finding (n = 4); 2 findings (n = 5); or ≥ 3 findings (n = 31). ND, no data.

**Figure 1:**
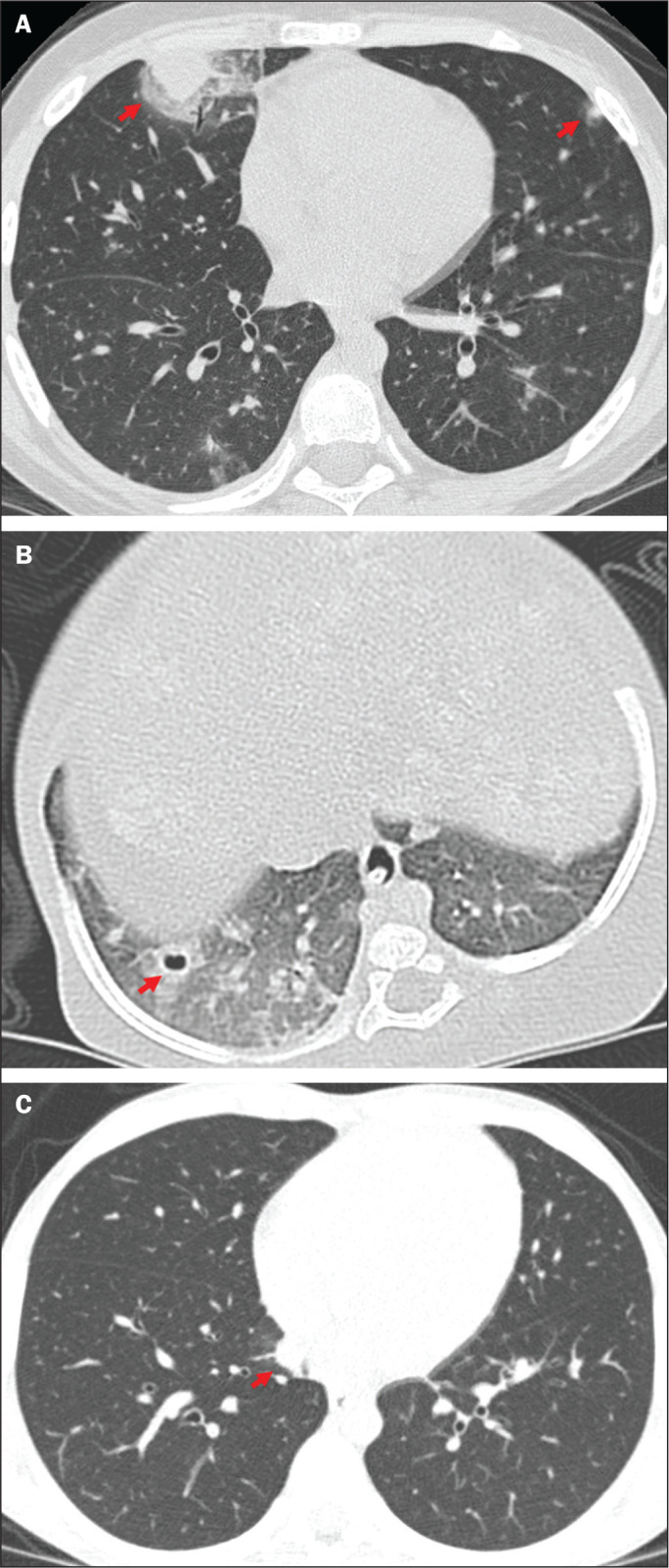
Findings typical of IFI on chest CT scans: multiple nodules with the halo sign (A); and cavities (B). C: Nodule without the halo sign (typical finding according to new guideline).

Among the 40 patients evaluated, radiological aspects considered typical of IFI in the 2008 EORTC guidelines^(18)^ were seen in 29 (72.5%): the halo sign, in 27 (67.5%); and one or more cavities, in seven (17.5%). The air crescent sign was not observed in any of the patients. Aspects considered typical of IFI in the 2020 EORTC guidelines^(10)^ were seen in ten additional patients (25.0%): nodules, in two (5.0%); and consolidation, in eight (20.0%). Atypical findings (pleural effusion and ground-glass opacities) were seen in only one patient (2.5%). Typical images are shown in Figure 1.

Follow-up chest CT scans were obtained between one week and three months after the initial scans, at the discretion of the treating physician. Among the 36 patients who underwent CT during the follow-up, improvement was shown in 21 (52.5%), worsening in 12 (30.0%), and no change in three (7.5%).

We compared the two groups of patients in which the CT findings were considered typical and atypical of IFI, respectively, in the 2008 EORTC guidelines^(18)^. The median age was higher in the former group than in the latter (123 vs. 77 months; *p* = 0.03), as was the number of days with neutropenia before the diagnosis (15 vs. 11; *p* = 0.04), whereas the proportion of patients with hemodynamic instability was higher in the latter group (54.5% vs. 20.6%; *p* = 0.03), as was the proportion of patients admitted to the intensive care unit (72.7% vs. 34.4%; *p* = 0.04). Other characteristics, such as the underlying condition, antibiotic use, antifungal prophylaxis, duration of treatment, and outcome, were similar between the two groups (Table 1).

Ten patients had proven IFIs—with *Aspergillus* sp. (n = 4); *Candida* sp. (n = 5); or *Fusarium* sp. (n = 1)—and one had a probable IFI (with *Aspergillus* sp.) according to the EORTC criteria^(10)^ (Table 2). In all five of the patients with proven or probable aspergillosis, the chest CT scan revealed the halo sign, which was also observed in one patient with disseminated candidiasis. The CT findings, by infectious etiology, are shown in Table 3. The remaining 29 patients were classified as having possible IFI. Clinical and demographic characteristics, as well as outcomes, were similar between the patients with proven/probable disease and those with possible disease.

**Table 2:** Demographic and clinical characteristics of patients with proven or probable IFI.

Patient	Sex	Age (years)	Underlying condition	IFI status	Fungal isolate	Site(s) of detection	Symptoms	CT findings
1	Male	6	Acute lymphoblastic leukemia	Proven	*Aspergillus* sp.	Lung (biopsy)	Fever and respiratory distress	Halo sign; ground-glass opacities; bronchial wall thickening
2	Male	14	Acute myeloid leukemia refractory or relapsed	Proven	*Aspergillus* sp.	Lung (biopsy)	Fever	Halo sign
3	Female	6	Acute myeloid leukemia	Proven	*Aspergillus* sp.	Lung (biopsy)	Fever, shock and respiratory distress	Halo sign; consolidation; pleural effusion; ground-glass opacities; mosaic attenuation pattern; bronchial wall thickening
4	Female	6	Acute myeloid leukemia	Proven	*Candida parapsilosis*	Central venous catheter	Fever and shock	Nodule
5	Female	1	Acute lymphoblastic leukemia	Proven	*Aspergillus* sp.	Lung (biopsy)	Fever and respiratory distress	Halo sign
6	Male	8	Neuroblastoma + autologous bone marrow transplant	Proven	*Candida haemulonii*	Central venous catheter; peripheral blood (culture)	Fever	Halo sign; cavity; bronchial wall thickening
7	Female	1	Hemangioendothe- lioma	Proven	*Candida parapsilosis*	Peripheral blood (culture); peritoneal cavity (ascitic fluid analysis)	Fever without neutropenia, shock and respiratory distress	Nodule; consolidation; cavity; ground- glass opacities; mosaic attenuation pattern; bronchial wall thickening
8	Female	5	Acute lymphoblastic leukemia refractory or relapsed	Proven	*Candida albicans*	Peripheral blood (culture); urine (culture); lung (bronchoalveolar lavage)	Fever, shock and respiratory distress	Consolidation; pleural effusion; ground-glass opacities; bronchial wall thickening
9	Female	11	Acute lymphoblastic leukemia refractory or relapsed	Proven	*Fusarium* sp.	Skin (biopsy)	Fever, respiratory distress and skin lesion	Consolidation; pleural effusion; ground-glass opacities
10	Male	3	Retinoblastoma	Proven	*Candida parapsilosis*	Peripheral blood (culture)	Fever, shock and respiratory distress	Consolidation; ground-glass opacities; bronchial wall thickening
11	Female	17	Acute lymphoblastic leukemia	Probable	*Aspergillus* sp.	Lung (bronchoalveolar lavage)	Fever and shock	Halo sign; consolidation; cavity; ground glass opacities; bronchial wall thickening

**Table 3:** Chest CT findings by infectious etiology.

CT finding*	Proven/probable aspergillosis (n = 5)	Proven candidiasis (n = 5)	Proven fusariosis (n = 1)	Possible IFI (n = 29)
Halo sign, n (%)	5 (100)	1 (20)	0 (0)	21 (72)
Consolidation, n (%)	2 (40)	3 (60)	1 (100)	14 (48)
Cavity, n (%)	2 (40)	2 (40)	0 (0)	3 (10)
Nodules, n (%)	0 (0)	2 (40)	0 (0)	1 (3)
Ground-glass opacity, n (%)	3 (60)	3 (60)	1 (100)	22 (75)
Pleural effusion, n (%)	1 (20)	1 (20)	1 (100)	12 (41)

* 1 finding (n = 4); 2 findings (n = 5); or ≥ 3 findings (n = 31).

Interobserver agreement varied depending on the clinical finding and improved after the second round of interpretation. As can be seen in Table 4, the level of interobserver agreement was high for pleural effusion (κ = 0.900; *p* < 0.001), cavities (κ = 0.860; *p* < 0.001), and ground-glass opacity (κ = 0.813; *p* < 0.001), whereas it was lower for nodules (κ = 0.649; *p* < 0.001), the halo sign (κ = 0.609; *p* < 0.001), and consolidation (κ = 0.609; *p* < 0.001). In the third round, the two radiologists jointly reviewed 18 CT scans and reached a consensus in all cases.

**Table 4:** Agreement between radiologists for chest CT findings.

Chest CT finding	Interobserver agreement			
	First round*		Second round^†^	
	Kappa	*P*-value	Kappa	*P*-value
Halo sign	0.609	< 0.001	0.762	< 0.001
Consolidation	0.609	< 0.001	0.921	< 0.001
Cavity	0.860	< 0.001	0.860	< 0.001
Nodules	0.649	< 0.001	0.782	< 0.001
Ground-glass opacity	0.813	< 0.001	0.865	< 0.001
Pleural effusion	0.900	< 0.001	0.966	< 0.001

* Review of all CT scans.

† Review of only the CT scans for which there were differences in interpretation in the first round.

## DISCUSSION

Although knowledge of the radiological findings typical of IFI is crucial for the early diagnosis in pediatric patients^(19)^, there have been few studies defining such findings in this population^(6-9,11,12,14-17)^. Guidelines suggest that even nonspecific findings should be considered relevant when pursuing a diagnosis of IFI^(10,19-21)^. Recent changes in the EORTC guidelines included the addition of other radiological patterns considered typical of IFI^(10)^. We found that 72.5% of our patients had findings typical of IFI according to the 2008 EORTC guidelines. There were ten patients (25.0%) who would not have been classified as having IFI on the basis of the 2008 guidelines but were included on the basis of the 2020 guidelines. Six of those ten patients recovered from the infection after treatment with an antifungal agent. That finding supports the change of classification.

Different frequencies of radiological patterns have been described in pediatric patients. In a study of 34 pediatric patients with IFI^(6)^, the halo sign was observed in 29%. A multicenter study of 139 patients with invasive aspergillosis showed that the halo sign was present in 10%^(8)^. In a study of pediatric patients with hematologic/oncologic diseases in Korea^(14)^, the halo sign was observed in 78% of the 37 patients with proven or probable aspergillosis and in 40% of the 228 with possible aspergillosis. In our sample of pediatric patients with proven, probable, or possible IFI, the halo sign was observed in 27 (67.5%)—in the initial CT scan in 24 patients and in the follow-up scan in three additional patients. That is a high rate in comparison with those reported in other studies of pediatric patients. Although the halo sign is considered to be an early finding in IFI^(22)^, another study conducted in Brazil suggested that ground-glass opacities and a tree-in-bud pattern can appear even earlier^(23)^. All of the patients in our sample for whom the halo sign was observed only on the follow-up CT scan had presented with other findings, including ground-glass opacities, in the initial scan. A review of 1,977 patients showed that the presence of the halo sign on a CT scan has a sensitivity of 54% and a specificity of 92% for the diagnosis of invasive aspergillosis^(24)^. The halo sign can be seen in other mold infections^(18,25,26)^, albeit less common in patients with invasive candidiasis^(25)^. In the present study, the halo sign was observed in all of the patients with proven or probable aspergillosis and in one patient infected with *Candida haemulonii*.

The air crescent sign is rare in children and is a marker of recovery that can usually be identified after two weeks of disease^(6,8,16,17)^. That sign was not observed in any of the patients in our sample.

In accordance with the findings of other studies^(8)^, we found that patients with the halo sign and cavities were, on average, older than were those with other findings (123 vs. 77 months; *p* = 0.03). That could be attributed to differences in host immune responses and delayed acquisition of CT scans^(26)^.

In our sample, the patients with nonspecific radiological findings had IFIs that were more severe, more often had hemodynamic instability, and were more likely to be admitted to the intensive care unit. That might be because those patients were younger or because the nonspecific presentation resulted in a delayed diagnosis.

Disagreements between radiologists in the interpretation of lung nodules have been reported in other studies^(27,28)^. In the present study, the level of interobserver agreement was higher than that reported previously^(27,28)^. That could be due to the fact that we did not measure the lesions, only classifying them as present or absent, thus increasing the likelihood of agreement. There are technical obstacles to obtaining appropriate CT scans in children, such as the need for sedation, the use of lower doses of radiation, and the presence of comorbidities that can affect the images. A study conducted in Australia showed a high rate of possible IFI in children, which was attributed to technical difficulties in image acquisition as well as to atypical findings on CT scans^(29)^. In our sample, the post-antifungal treatment outcomes for the group with halo signs and cavities were similar to those observed for the group with other findings, indicating that all new radiological images should be taken into consideration in pediatric patients with risk factors for IFI^(19)^.

Our study has several limitations, including the small number of patients and its retrospective nature. The fact that galactomannan and beta-D-glucan were not routinely measured is a limitation because it prevented us from determining the true number of patients with probable (rather than just possible) disease. In addition, the fact that the patients were not routinely sedated before undergoing chest CT could explain why some of the scans had minor technical defects.

## CONCLUSION

We found that the incidence of chest CT findings typical of IFI was 72.5% when we applied the 2008 EORTC guidelines and 97.5% when we applied the 2020 version of those guidelines. The recent changes in the classification of findings indicative of IFI on chest CT scans have made it possible to diagnose more patients with IFI. Therefore, a diagnosis of IFI should be considered in children and adolescents with relevant risk factors and any new abnormalities on chest CT scans.

## References

[1] Pagano L, Caira M, Nosari A (2007). Fungal infections in recipients of hematopoietic stem cell
transplants: results of the SEIFEM B-2004 Study—Sorveglianza Epidemiologica
Infezioni Fungine Nelle Emopatie Maligne. Clin Infect Dis.

[2] Sung L, Lange BJ, Gerbing RB (2007). Microbiologically documented infections and infection-related
mortality in children with acute myeloid leukemia. Blood.

[3] Rubio PM, Sevilla J, González-Vicent M (2009). Increasing incidence of invasive aspergillosis in pediatric
hematology oncology patients over the last decade. J Pediatr Hematol Oncol.

[4] Zaoutis TE, Heydon K, Chu JH (2006). Epidemiology, outcomes, and costs of invasive aspergillosis in
immunocompromised children in the United States, 2000. Pediatrics.

[5] Kobayashi R, Kaneda M, Sato T (2008). The clinical feature of invasive fungal infection in pediatric
patients with hematologic and malignant diseases: a 10-year analysis at a
single institution at Japan. J Pediatr Hematol Oncol.

[6] Georgiadou SP, Pongas G, Fitzgerald NE (2012). Invasive mold infections in pediatric cancer patients reflect
heterogeneity in etiology, presentation, and outcome: a 10-year,
single-institution, retrospective study. J Pediatric Infect Dis Soc.

[7] Ozsevik SN, Sensoy G, Karli A (2015). Invasive fungal infections in children with hematologic and
malignant diseases. J Pediatr Hematol Oncol.

[8] Burgos A, Zaoutis TE, Dvorak CC (2008). Pediatric invasive aspergillosis: a multicenter retrospective
analysis of 139 contemporary cases. Pediatrics.

[9] Hasan RA, Abuhammour W (2006). Invasive aspergillosis in children with hematologic
malignancies. Pediatr Drugs.

[10] Donnelly JP, Chen SC, Kauffman CA (2020). Revision and update of the consensus definitions of invasive
fungal disease from the European Organization for Research and Treatment of
Cancer and the Mycoses Study Group Education and Research
Consortium. Clin Infect Dis.

[11] Katragkou A, Fisher BT, Groll AH (2017). Diagnostic imaging and invasive fungal diseases in
children. J Pediatric Infect Dis Soc.

[12] Toma P, Bertaina A, Costagrola E (2016). Fungal infections of the lung in children. Pediatr Radiol.

[13] Lehrnbecher T, Becker K, Groll AH (2017). Current algorithms in fungal diagnosis in the immunocompromised
host. Methods Mol Biol.

[14] Han SB, Kim SK, Bae EY (2015). Clinical features and prognosis of invasive pulmonary
aspergillosis in Korean children with hematologic/oncologic
diseases. J Korean Med Sci.

[15] Steinbach WJ (2010). Invasive aspergillosis in pediatric patients. Curr Med Res Opin.

[16] Taccone A, Occhi M, Garaventa A (1993). CT of invasive pulmonary aspergillosis in children with
cancer. Pediatr Radiol.

[17] Thomas KE, Owens CM, Veis PA (2003). The radiological spectrum of invasive aspergillosis in children:
a 10-year review. Pediatr Radiol.

[18] De Pauw B, Walsh TJ, Donnelly JP (2008). Revised definitions of invasive fungal disease from the European
Organization for Research and Treatment of Cancer/Invasive Fungal Infections
Cooperative Group and the National Institute of Allergy and Infectious
Diseases Mycoses Study Group (EORTC/MSG) Consensus Group. Clin Infect Dis.

[19] Lehrnbecher T, Hassler A, Groll AH (2018). Diagnostic approaches for invasive aspergillosis-specific
considerations in the pediatric population. Front Microbiol.

[20] Steinbach WJ (2005). Pediatric aspergillosis: disease and treatment differences in
children. Pediatr Infect Dis J.

[21] Carlesse F, Daudt LE, Seber A (2019). A consensus document for the clinical management of invasive
fungal diseases in pediatric patients with hematologic cancer and/or
undergoing hematopoietic stem cell transplantation in Brazilian medical
centers. Braz J Infect Dis.

[22] Caillot D, Couaillier JF, Bernard A (2001). Increasing volume and changing characteristics of invasive
pulmonary aspergillosis on sequential thoracic computed tomography scans in
patients with neutropenia. J Clin Oncol.

[23] Nucci M, Nouér SA, Cappone D (2013). Early diagnosis of invasive pulmonary aspergillosis in
hematologic patients: an opportunity to improve the outcome. Haematologica.

[24] Ray A, Mittal A, Vyas S (2020). CT halo sign: a systematic review. Eur J Radiol.

[25] Althoff Souza C, Müller NL, Marchiori E (2006). Pulmonary invasive aspergillosis and candidiasis in
immunocompromised patients: a comparative study of the high-resolution CT
findings. J Thorac Imaging.

[26] Georgiadou SP, Sipsas NV, Marom EM (2011). The diagnostic value of halo and reversed halo signs for invasive
mold infections in compromised hosts. Clin Infect Dis.

[27] Armato SG, McNitt-Gray MF, Reeves AP (2007). The Lung Image Database Consortium (LIDC): an evaluation of
radiologist variability in the identification of lung nodules on CT
scans. Acad Radiol.

[28] Leader JK, Warfel TE, Fuhrman CR (2005). Pulmonary nodule detection with low-dose CT of the lung:
agreement among radiologists. AJR Am J Roentgenol.

[29] Wang SS, Kotecha RS, Bernard A (2019). Invasive fungal infections in children with acute lymphoblastic
leukaemia: results from four Australian centres, 2003-2013. Pediatr Blood Cancer.

